# Supplementary material for: Immunogenetic Profile Associated with Patients Living with HIV-1 and Epstein–Barr Virus (EBV) in the Brazilian Amazon Region
Source: Viruses. 2024 Jun 24;16(7):1012. doi: 10.3390/v16071012 (PMC11281405; doi:10.3390/v16071012)
Supplement: Supplementary file 1 [file viruses-16-01012-s001.zip › viruses-3024448-supplementary.pdf]

Table S1. Comparative analysis between genotypes of SNPs studied and plasma concentration of cytokines IL-2, IL-4, IL-6, TNF-α, and IFN-γ using Kruskal-Wallis.

ID	Gene	Cytokine	Groups	*p-value*
	IL2	IL-2	G1	0.59
rs2069762			G2	**0.04**
			G3	0.10
			G4	0.55
rs2243250	*IL4*	IL-4	G1	0.76
			G2	0.51
			G3	0.49
			G4	**0.04**
rs1800795	*IL6*	IL-6	G1	0.83
			G2	0.20
			G3	0.26
			G4	0.22
rs1800796	*IL6*	IL-6	G1	0.10
			G2	0.57
			G3	0.39
			G4	0.13
rs1799964	*TNF*	TNF- α	G1	0.40
			G2	0.87
			G3	0.10
			G4	0.55
rs2069705	*IFNG*	IFN-γ	G1	0.15
			G2	0.91
			G3	0.10
			G4	**0.03**

Table S2. Comparative Analysis between genotypes of SNPs studied and CD4+ T lymphocyte counts using ANOVA test.

ID	Gene	Groups	F	*p-value*
	IL2	G1	0.19	0.83
rs2069762		G2	0.56	0.57
		G3	0.42	0.52
		G4	0.30	0.74
rs2243250	*IL4*	G1	0.78	0.46
		G2	0.43	0.65
		G3	0.41	0.67
		G4	0.03	0.97
rs1800795	*IL6*	G1	2.61	0.08
		G2	0.39	0.68
		G3	0.38	0.69
		G4	0.16	0.85
rs1800796	*IL6*	G1	0.30	0.74
		G2	0.30	0.74
		G3	0.35	0.70
		G4	0.32	0.73
rs1799964	*TNF*	G1	2.42	0.10
		G2	0.52	0.60

		G3	0.48	0.10
		G4	0.61	0.55
rs2069705	*IFNG*	G1	0.02	0.98
		G2	4.06	**0.02**
		G3	0.12	0.52
		G4	2.81	0.06

Table S3. Comparative Analysis between genotypes of SNPs studied and CD8+ T Lymphocyte Counts using ANOVA test.

**ID**	**Gene**	**Groups**	**F**	***p-value***
	IL2	G1	1.36	0.26
rs2069762		G2	0.05	0.95
		G3	0.10	0.85
		G4	0.62	0.54
rs2243250	*IL4*	G1	2.95	0.06
		G2	0.95	0.39
		G3	1.99	0.15
		G4	1.29	0.28
rs1800795	*IL6*	G1	1.28	0.28
		G2	0.26	0.77
		G3	0.37	0.69
		G4	0.11	0.89
rs1800796	*IL6*	G1	0.80	0.56
		G2	1.88	0.15
		G3	1.16	0.33
		G4	5.65	**<0.01**
rs1799964	*TNF*	G1	1.69	0.19
		G2	4.33	**0.01**
		G3	1.02	0.10
		G4	0.41	0.66
rs2069705	*IFNG*	G1	1.30	0.28
		G2	0.94	0.39
		G3	1.01	0.05
		G4	3.15	**0.04**

Table S4. Comparative analysis of studied genotypes of SNPs and HIV viral load using the Kruskall-Wallis test.

**ID**	**Gene**	**Groups**	***p-value***
rs2069762	*IL2*	G1	0.53
		G2	0.86
rs2243250	*IL4*	G1	0.44
		G2	0.84
rs1800795	*IL6*	G1	0.70
		G2	0.06
rs1800796	*IL6*	G1	0.10
		G2	0.25
rs1799964	*TNF*	G1	0.78
		G2	0.68
rs2069705	*IFNG*	G1	0.70
		G2	0.06
